# Vulvar Leiomyoma Masquerading as Bartholin Cyst: A Case Report of Unveiled Diagnosis and Surgical Management

**DOI:** 10.7759/cureus.47845

**Published:** 2023-10-27

**Authors:** Nandini Komatineni, Krishna Gopal Paul

**Affiliations:** 1 Obstetrics and Gynaecology, Varun Arjun Medical College and Rohilkhand Hospital, Shahjahanpur, IND

**Keywords:** surgical management, histopathological diagnosis, painless vulval mass, bartholin cyst, vulvar leiomyoma

## Abstract

Vulvar leiomyomas are rare benign tumors originating from smooth muscle cells of the vulvar tissue. We present the case of a 44-year-old female patient complaining of a painless vulval mass for 12 years, gradually increasing from 1x1 cm to 5x4 cm. Clinical assessment initially suggested a Bartholin cyst because of its non-tender and non-fluctuant nature. However, surgical intervention revealed an unexpected diagnosis of vulvar leiomyoma, measuring 5x5x4 cm. The patient underwent successful excision and repair under spinal anesthesia. This case underscores the significance of meticulous clinical evaluation and accurate histopathological examination in distinguishing vulvar masses. Accurate diagnosis guides appropriate management, and long-term follow-up prevents complications and recurrence. This report highlights the diagnostic challenges of rare vulvar lesions and the importance of a comprehensive approach to their evaluation and treatment.

## Introduction

Vulvar leiomyomas are notably rare among gynecologic neoplasms and vulvar tumors, originating from the smooth muscle cells within the vulvar tissue [1]. While they represent a distinct subset within this category, it is essential to emphasize their infrequency in clinical practice. The rarity of vulvar leiomyomas is underscored by their relatively limited occurrence, especially compared to more common vulvar conditions and other gynecologic neoplasms. To put this into perspective, vulvar leiomyomas are distinctly uncommon compared to other gynecologic neoplasms and vulvar tumors. This rarity is exemplified by the fact that these tumors arise from muscular components of the vulva and are considered a unique entity within the broader category of uterine leiomyomas. Hence, their prevalence in clinical settings is limited, and they are seldom encountered by healthcare providers [1].

In contrast, clinicians often encounter more prevalent vulvar conditions, such as Bartholin cysts. Bartholin cysts, characterized by localized vulvar swelling, exhibit a significantly higher incidence compared to vulvar leiomyomas [2]. The clinical presentation of vulvar leiomyomas can, at times, mimic conditions like Bartholin cysts, which further highlights the diagnostic challenges that clinicians face when confronted with these rare tumors [3]. This discrepancy in prevalence becomes evident when comparing the incidence rates of vulvar leiomyomas to those of more common gynecologic neoplasms and vulvar conditions, as illustrated in the existing literature.

Bartholin cysts are typically painless, subcutaneous swellings located near the posterior aspect of the labia majora [4]. Their etiology involves the obstruction of the Bartholin gland duct, accumulating fluid, and glandular secretions. Because of the painless nature of both vulvar leiomyomas and Bartholin cysts, a misdiagnosis can occur, delaying proper management and potentially affecting patient outcomes [5].

Accurate diagnosis is crucial for guiding appropriate treatment decisions. Vulvar leiomyomas, albeit rare, require a distinct approach, often involving surgical excision [6]. The surgical management of vulvar lesions requires meticulous planning to ensure complete removal and optimal cosmetic outcomes, especially in the sensitive vulvar region. Misdiagnosing a vulvar leiomyoma as a Bartholin cyst can lead to unnecessary interventions or inadequate treatment. Therefore, the differentiation between these entities is essential to offer tailored and effective care [7].

In this case report, we present a patient who initially presented with a painless vulval mass clinically diagnosed as a Bartholin cyst because of its non-tender and non-fluctuant nature. Subsequent surgical exploration revealed a surprising histopathological diagnosis of vulvar leiomyoma. This report emphasizes the importance of accurate clinical assessment, histopathological evaluation, and appropriate surgical intervention for managing vulvar lesions. Through this case, we highlight the complexities of diagnosing rare vulvar conditions and underscore the need for a comprehensive approach to their assessment and treatment.

## Case presentation

**Figure 1:**
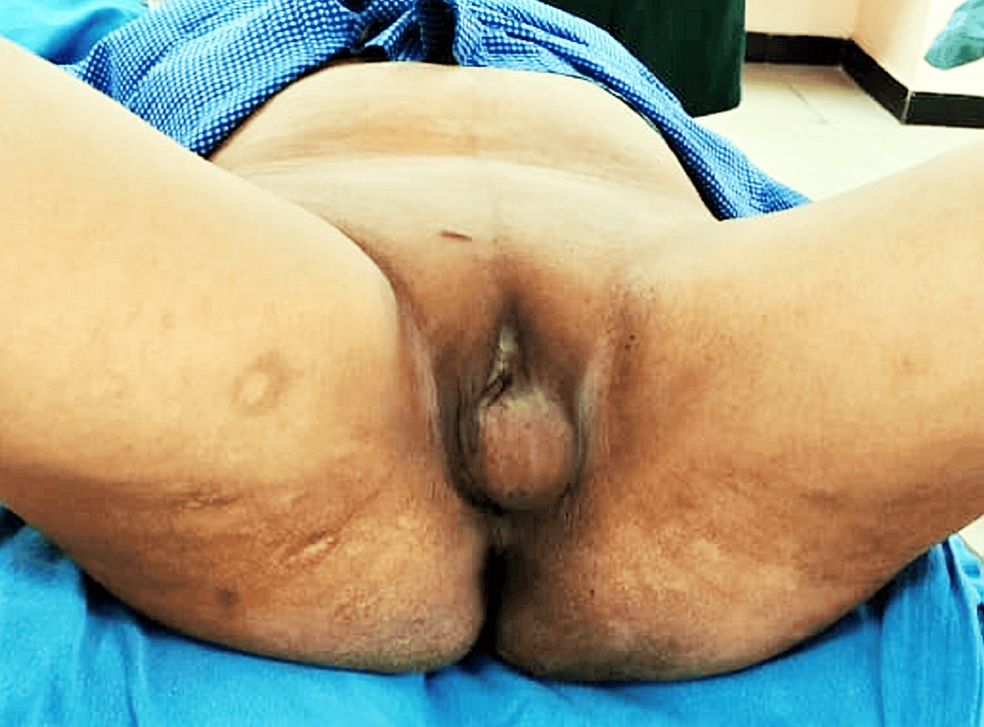
Lithotomy position showing a left vulval mass measuring 5×5×4 cm

We present the case of a 44-year-old female patient who presented with a longstanding complaint of a painless vulval mass. The mass had been present for 12 years and had gradually increased from an initial measurement of 1x1 cm to the current dimensions of 5x4 cm (Figure 1). The patient had no significant medical, family, or psychosocial history. She had not undergone any past interventions or surgeries.

Upon physical examination, the patient was found to be in good general health, with stable vital signs, including a pulse rate of 88 beats per minute, blood pressure of 110/70 mmHg, respiratory rate of 12 cycles per minute, temperature of 98°F, and oxygen saturation of 98% on room air. Cardiovascular examination revealed the presence of S1 and S2 heart sounds without murmurs or added sounds. Respiratory examination showed bilateral normal vesicular breath sounds with no added sounds. The patient was conscious, alert, and oriented to time, place, and person.

An abdominal examination revealed a soft, non-tender abdomen with no organomegaly. Perineal examination revealed a localized swelling on one side of the vulva, occupying the posterior half of the labia majora. The swelling measured 5x4 cm and exhibited thinned and shiny skin Figure 1. The cyst was non-tender and non-fluctuant, leading to a clinical diagnosis of Bartholin cyst.

**Figure 2:**
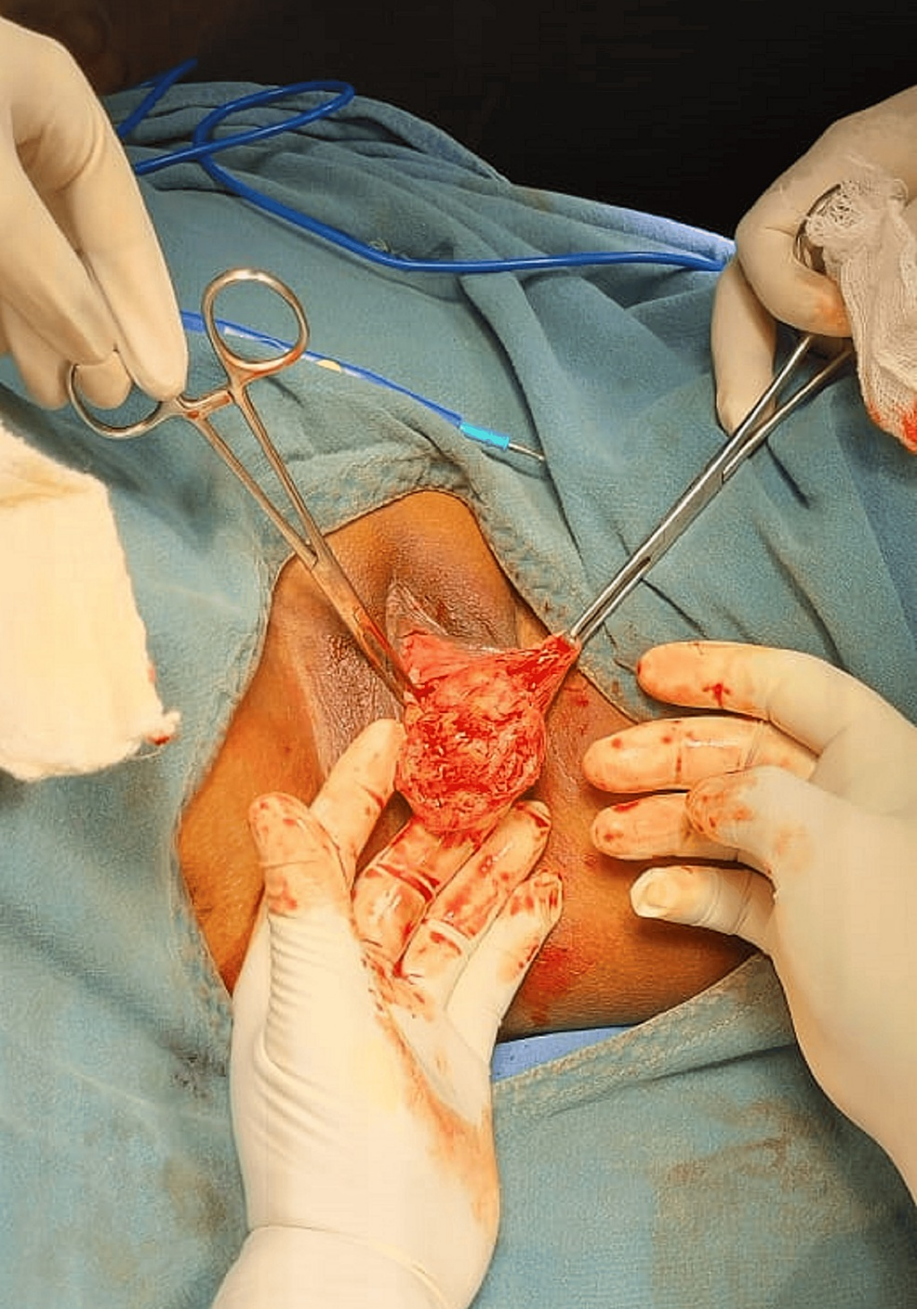
Operative picture showing excision of mass

**Figure 3:**
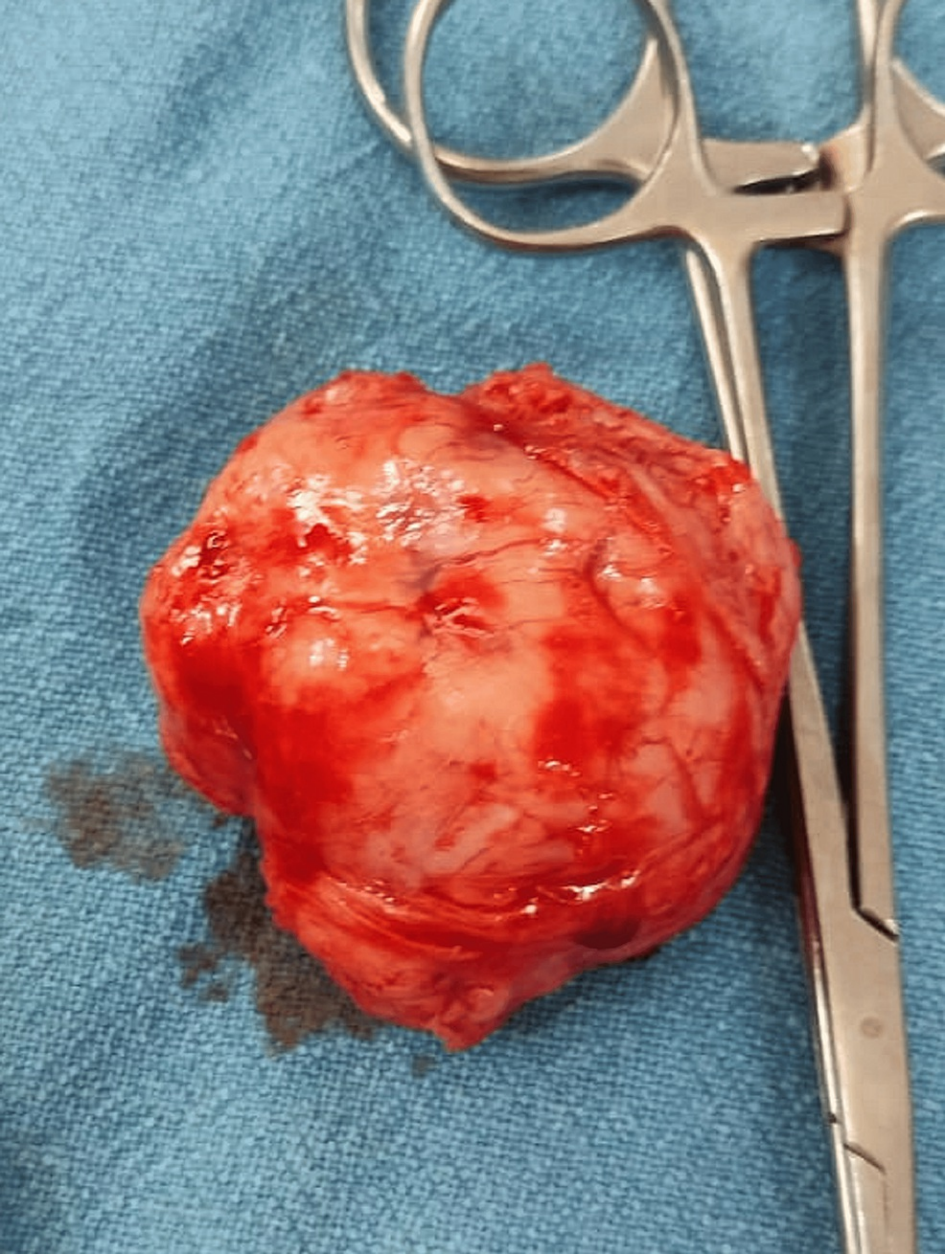
Postoperative picture showing a specimen of the excised mass

The patient underwent an incision and repair procedure under spinal anesthesia, which was uneventful (Figure 2). The histopathology report revealed a surprising finding - the excised mass was diagnosed as a vulvar leiomyoma measuring 5x5x4 cm (Figure 3). Regarding the patient's postoperative progress, we would like to provide further information on her follow-up status. Following the surgical excision of the vulvar leiomyoma, the patient experienced a smooth recovery with no immediate complications. She was discharged in a satisfactory condition on the third postoperative day. Notably, during the subsequent follow-up visits, the patient continued to exhibit an uneventful recovery, with no significant postoperative complications or adverse events reported.

**Figure 4:**
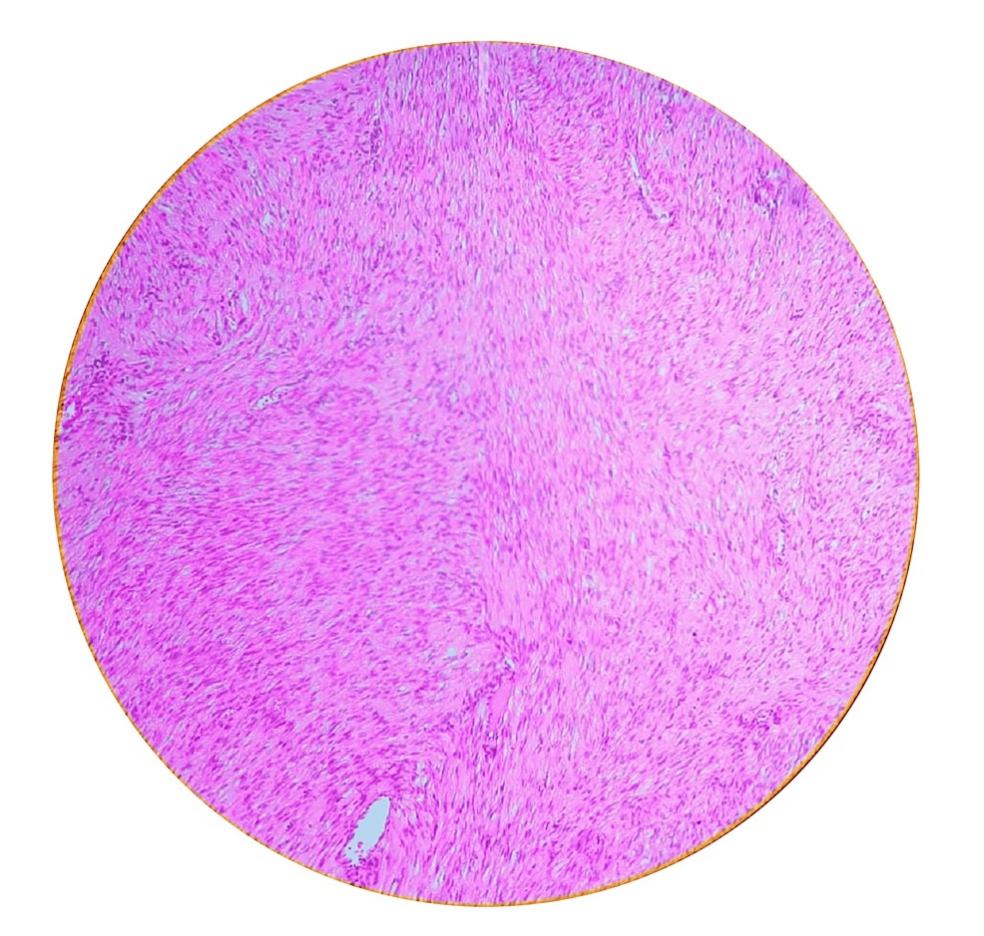
Histopathology hematoxylin and eosin stain 100x showing bundles of benign-appearing spindle cells with oval to spindle shapes nuclei along with eosinophilic cytoplasm

Histopathologically examining the excised vulvar mass using hematoxylin and eosin (H&E) staining revealed a distinctive tissue architecture. The specimen exhibited a pattern of benign-appearing spindle cells organized into interacting bundles. These spindle cells displayed oval to spindle-shaped nuclei and eosinophilic cytoplasm, consistent with the characteristic features of leiomyoma cells. Furthermore, intervening fibrous septa and dilated, congested vessels within the tissue were noted. Notably, no evidence of malignancy was observed in the sections examined, which confirms the benign nature of the vulvar leiomyoma. These histopathological findings align with the clinical diagnosis and concord with the immunohistochemical results, collectively supporting accurately characterizing the lesion as a benign vulvar leiomyoma (Figure 4).

**Figure 5:**
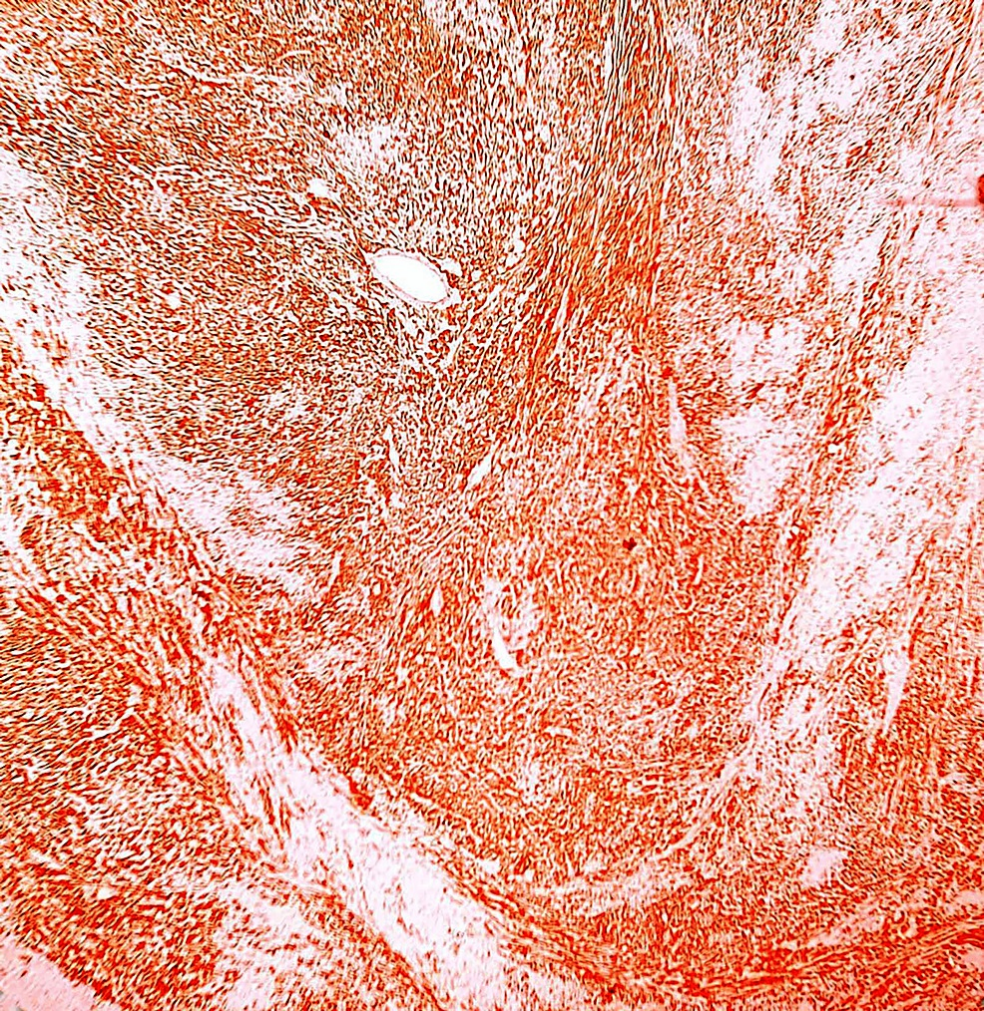
Desmin shows diffuse and strong positive results, confirming smooth muscle origin of the tumor

**Figure 6:**
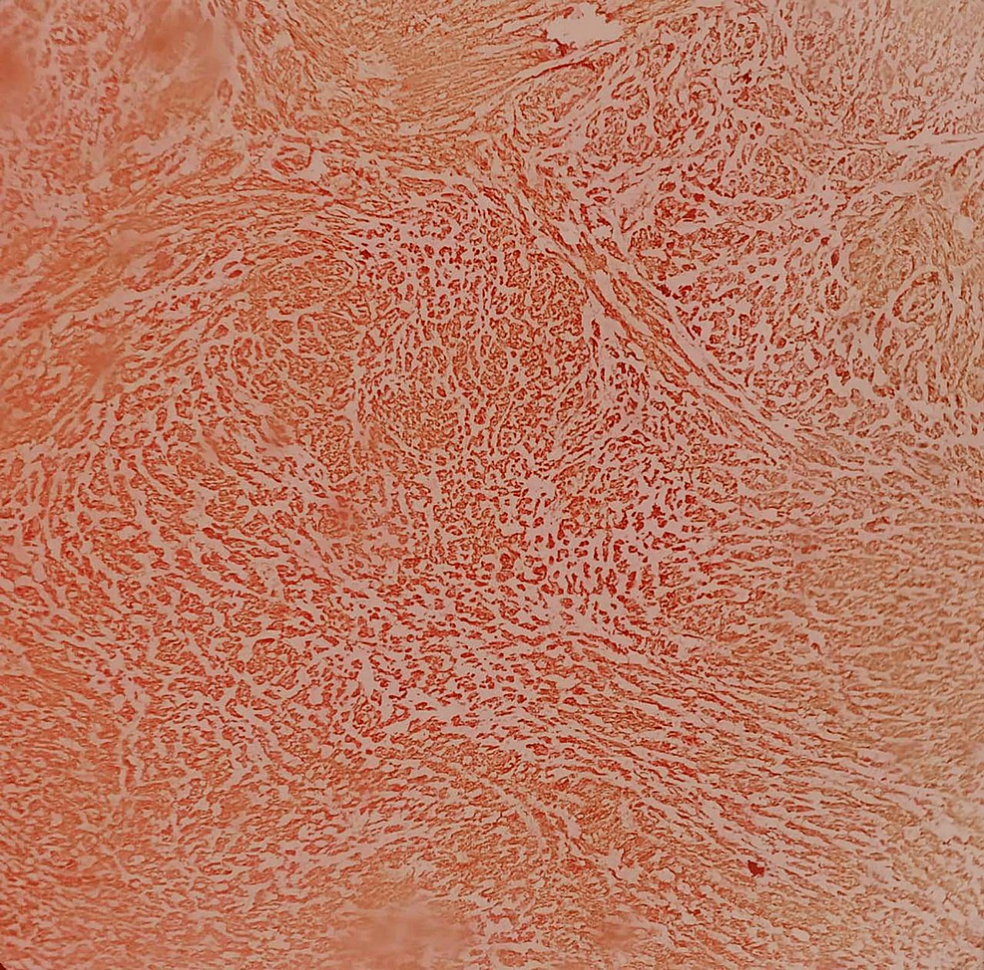
Smooth muscle actin shows diffuse and strong positive results with the leiomyomatous nature of the lesion

**Figure 7:**
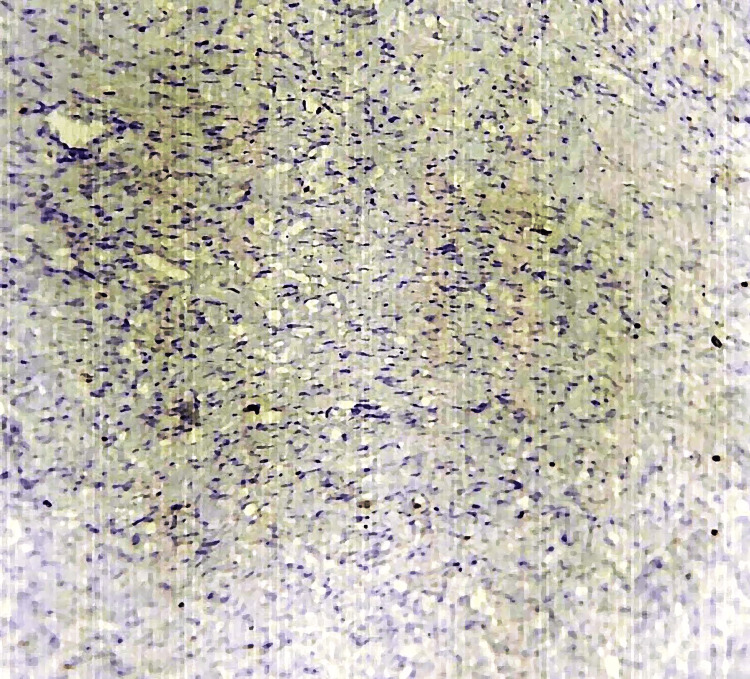
Ki67 proliferative index was shallow, measuring less than 1%, indicating a low cellular proliferation rate

An immunohistochemistry analysis of the excised vulvar leiomyoma revealed notable findings. Desmin staining exhibited diffuse and strong positivity (Figure 5), confirming the smooth muscle origin of the tumor. Similarly, smooth muscle actin staining also showed diffuse and strong positive results, consistent with the leiomyomatous nature of the lesion (Figure 6). Notably, the Ki67 proliferative index was shallow, measuring less than 1%, indicating a low cellular proliferation rate and suggesting the tumor’s benign nature (Figure 7). These immunohistochemical findings further support the diagnosis of a benign vulvar leiomyoma and are consistent with the histopathological evaluation of the excised mass.

Routine investigations, including coagulation profile, liver and renal function tests, chest X-ray, and ECG were all within normal limits. Furthermore, an abdominal and pelvic ultrasound showed no abnormalities. This case underscores the importance of accurate clinical evaluation and histopathological diagnosis in managing vulvar masses. Vulvar leiomyomas are relatively uncommon, and their presentation can mimic other vulvar conditions, as evidenced by the initial clinical diagnosis of a Bartholin cyst. Long-term follow-up may be recommended to monitor the patient's condition and detect potential recurrences or complications.

## Discussion

The presented case of a 44-year-old female patient with a longstanding painless vulval mass highlights the challenges in diagnosing and managing rare vulvar lesions. Based on the patient's history and physical examination, the initial clinical diagnosis of a Bartholin cyst underscores the importance of considering a broad differential diagnosis for vulvar masses. Moreover, this case report emphasizes the significance of accurate clinical evaluation and histopathological diagnosis in guiding appropriate management strategies. Vulvar masses encompass a wide range of benign and malignant conditions, each with distinct clinical and histopathological characteristics (Figure 4). Accurately identifying these lesions is essential for providing appropriate care and preventing mismanagement [8-9]. In this case, the patient's long-standing history of a painless vulval mass raises questions about the delay in seeking medical attention and the potential impact of such delays on diagnosis and treatment outcomes.

While vulvar leiomyomas are rare and benign smooth muscle tumors that can also manifest in extragenital sites, such as the vulva [10], it is essential to consider a broader spectrum of potential differential diagnoses, particularly when presented with vulvar masses. The initial clinical misdiagnosis of this case as a Bartholin cyst underscores the importance of distinguishing vulvar leiomyomas from other more common vulvar lesions. Evaluating vulvar masses should encompass a comprehensive assessment that includes distinguishing characteristics from conditions such as Bartholin cysts and other vulvar neoplasms. This emphasizes the need for a vigilant and multifaceted diagnostic approach [11]. This approach ensures accurate differentiation, appropriate management, and optimal patient care.

Histopathological examination played a crucial role in establishing the correct diagnosis in this case. The unexpected finding of a vulvar leiomyoma after surgical excision of the mass emphasizes the limitations of clinical diagnosis alone [12]. Histopathology confirmed the lesion’s nature and provided essential information about its size, cellular composition, and potential for malignancy. Notably, preoperative imaging, such as ultrasound, did not reveal the true nature of the mass, which further emphasizes the importance of histopathology [13].

The successful surgical management of the patient's vulvar leiomyoma underscores the importance of prompt intervention, especially when addressing enlarging masses or masses that present diagnostic challenges. Surgical excision, as performed in this case, is the primary treatment for symptomatic or enlarging vulvar leiomyomas. However, the rarity of these tumors highlights the limited evidence available regarding their optimal management, postoperative follow-up, and long-term outcomes [14]. Long-term follow-up is essential to monitor for potential recurrences or complications. Given the paucity of data on vulvar leiomyomas, continued observation and documentation of the patient's progress could contribute to a better understanding of the natural history of these lesions. Additionally, sharing such cases through publications contributes to the medical literature and can guide future clinical decisions [15].

This case report emphasizes the challenges in diagnosing and managing rare vulvar lesions (e.g., vulvar leiomyomas). Accurate clinical assessment and histopathological diagnosis guide appropriate management and prevent misdiagnosis. This case serves as a reminder of the diverse differential diagnoses for vulvar masses and underscores the importance of a multidisciplinary approach to patient care. Further research and long-term follow-up are needed to enhance our understanding of these uncommon vulvar neoplasms and optimize their management strategies.

## Conclusions

The presented case highlights the diagnostic intricacies associated with rare vulvar conditions, precisely the challenge of distinguishing between vulvar leiomyomas and Bartholin cysts. Accurate clinical evaluation is paramount, as these lesions share standard features, including painless presentation and localized swelling. The case underscores histopathological confirmation's significance in preventing misdiagnosis and ensuring appropriate management. The management of vulvar lesions demands tailored approaches to achieve optimal outcomes. Surgically excising vulvar leiomyomas requires careful planning because of their location and potential impact on cosmetic and functional aspects. A misdiagnosis of these lesions can lead to unnecessary interventions or inadequate treatment. This report highlights the importance of a comprehensive diagnostic approach involving clinical assessment and histopathological examination to differentiate between similar vulvar presentations. This distinction has crucial implications for patient management, emphasizing the significance of accurate diagnosis in providing effective and patient-centered care. The case underscores clinicians' need for ongoing vigilance and awareness when encountering rare vulvar lesions, ensuring that patients receive the most appropriate interventions.
